# Is the gastric tube a burden for the noninvasive ventilation?

**DOI:** 10.1186/cc14300

**Published:** 2015-03-16

**Authors:** I Minev, C Stefanov

**Affiliations:** 1 University Hospital 'St. George', Plovdiv, Bulgaria

## Introduction

The application of noninvasive ventilation (NIV) in ICUs has spread widely during the years. It is used in the treatment of different forms of acute respiratory failure and COPD exacerbations. Although NIV is thought to be more comfortable for patients than invasive mechanical ventilation, its failure rates in the ICUs range between 10 and 40%. Except for the interface-related problems, there are some specific considerations for the patient-ventilator interaction and the applied mechanical forces. During NIV there is a predisposition for the stomach to be inflated with gas, which could cause severe respiratory complications, especially in COPD patients, and thus prolong the mechanical ventilation and the weaning process. This remains one of the major causes for NIV failure. Although a lot of face masks with different interfaces are available on the market, just a few have additional ports for a NGT. They are characterized by higher price and a complex setup. In order to perform NIV in patients, requiring NGT placement, without additional air leaks and to be able to ensure their enteral nutrition and/or stomach drainage, we installed a port for a NGT on a standard face mask.

## Methods

In this study, six of the COPD patients admitted to our ICU, who required NGT placement, were ventilated with the Draeger Evita 2 dura through a modified reusable silicone face mask (UMDNS code: 12-453 with 22 mm ID connection; sizes 4 and 5) with silicone headgear and a hook ring. All of them had a NGT during their stay in the ICU. We evaluated the efficacy of our modification comparing the achieved Vt with modified and unmodified face mask, during two periods of 10 minutes. The mode and parameters of ventilation were not changed. We assessed patient comfort with a visual analogue scale.

## Results

The average duration of NIV was 3.5 days (SD = 1.6). We examined two sets of 10 consequent breathing cycles for every patient. The mean Vt was 472 ml (SD = 76 ml) with standard face mask and 460 ml (SD = 86 ml) with the modified one. There was statistically significant correlation between the two datasets (*P *< 0.05). No additional leaks were detected. According to the VAS evaluation, five of the patients (83%) had comfort improvement with the modified mask.

## Conclusion

With this modification of the face mask we achieved adequate drainage of the stomach and/or the enteral nutrition of the patients and improvement in their comfort during NIV, compared with the ventilation with a standard mask, without additional air leaks and at a low cost.

